# Runners with lower dynamic stability exhibit better running economy

**DOI:** 10.1038/s41598-025-26008-x

**Published:** 2025-10-31

**Authors:** Carlo von Diecken, Marlene Riedl, Steffen Willwacher, Olaf Ueberschär

**Affiliations:** 1 Department of Engineering and Industrial Design, Magdeburg-Stendal University of Applied Sciences, 39114 Magdeburg, Germany; 2 Institute for Advanced Biomechanics and Motion Studies, Offenburg University, 77652 Offenburg, Germany; 3 Department of Orthopaedic and Trauma Surgery, Martin Luther University Halle-Wittenberg, 06120 Halle, Germany; 4 Institute for Applied Training Science, 04109 Leipzig, Germany

**Keywords:** Running, Stability, Dynamic stability, Lyapunov, Biomechanics, Running economy, Engineering, Physiology

## Abstract

Maintaining dynamic stability during running incurs an energetic cost that does not contribute to forward propulsion. Despite this, dynamic stability has received little attention as a potential factor influencing running economy. To understand the relationship between dynamic stability and running economy, nineteen trained runners were tested on a treadmill across three individualized speeds. Whole-body dynamic stability was quantified via a single maximum Lyapunov exponent (MLE) computed from a 21D state-space embedding, which incorporated 3D angular velocities from seven body segments (bilateral: shank, upper torso, forearm; unilateral: lower torso) and running economy was measured as cost of transport (COT) using metabolic gas-exchange data. Linear mixed-effects models were used to assess the relationship between MLE and COT as well as the effects of running speed on MLE. MLE was negatively associated with COT (*p =* 0.049), while running speed had no significant effect on MLE (*p >* 0.579). This study is the first to demonstrate that the MLE calculated from a multivariate state-space is negatively associated with COT, indicating that runners with lower dynamic stability exhibit better running economy. Further, MLE was not affected by running speed, indicating that this measure of whole-body dynamic stability can be robustly assessed at a range of running speeds. These results may hint at a previously unexplored avenue to improve running economy through alteration of dynamic stability characteristics.

**Supplementary Information:**

The online version contains supplementary material available at 10.1038/s41598-025-26008-x.

## Introduction

Maintaining frontal plane stability is responsible for ~ 2% of overall energy cost of running^1^, with maintenance of sagittal and transverse plane stability likely incurring additional energy costs. Thus, running economy–the amount of oxygen or energy consumed per kilogram of bodyweight while running–cannot be comprehensively understood without considering dynamic stability. Despite this, dynamic stability has received little attention in running economy research^2^.

Recently, non-linear analysis methods have gained popularity as a means of quantifying dynamic stability during running^3^, capturing aspects of running biomechanics that may remain overlooked when quantifying stability through traditional linear measures derived from spatiotemporal or kinematic parameters^4^. For instance, the maximum short-time Lyapunov exponent (MLE) quantifies the average rate of divergence of initially neighboring trajectories in reconstructed state-space, reflecting the deterministic chaos inherent in complex, multi-degree-of-freedom systems. This measure, commonly interpreted as local dynamic stability (LDS)^5^, can be assessed with as little equipment as a single inertial measurement unit (IMU)^6,7^, making it well-suited to elucidate the relationship between stability and running economy in both laboratory and in-field conditions. To date, however, the connection between LDS and running economy has not been explicitly tested.

Nevertheless, previous work on LDS in running provides indirect evidence that the two may be related. Hoenig et al.^4^ and Frank et al.^8^ found that competitive and trained runners exhibit greater LDS than recreational runners, a finding that aligns with the superior running economy of the former populations^2^. This finding suggests that there might be an association between greater LDS and improved running economy. However, Hoenig et al.^4^ also reported that LDS increases during an exhaustive 5000 m run, which coincides with a worsening of running economy as evident by the V̇O₂ slow component^9^ and indicates that greater LDS may not necessarily be beneficial. Additionally, there are two studies that directly addressed the link between dynamic stability and running economy using linear and non-linear stability metrics other than LDS. While Schütte et al.^10^ found evidence that greater instability was associated with higher energy cost of running in recreational runners, Panday et al.^11^ reported that reduced stability may actually enable professional runners to run more efficiently than novice runners. These contradictory results highlight the need for more high-quality research to elucidate the seemingly complex relationship between dynamic stability and running economy.

Further complicating this issue is the fact that non-linear dynamic stability measures are often only assessed at a single joint or body segment, or include only a singular plane of motion^3,8,11,12^, which may not fully capture the stability of the multi-segment, three-dimensional system that is human running. It is, therefore, conceivable that clearer trends might emerge if dynamic stability was computed from three dimensional signals assessed at multiple segments of the whole body. In addition, MLE of specific joints and segments has been previously shown to be running speed–dependent, with both increases^13^ and decreases^14,15^ in stability reported with increasing running speed. It is currently unclear whether an MLE value that is derived from a multivariate state-space embedding that incorporates multiple body segments would also display running speed–dependent changes.

With the present study, we aim to investigate the relationship between dynamic stability and running economy in a sample of trained male and female runners during treadmill running at three individualized speeds. We further aim to determine whether an MLE value that is derived from a multivariate state-space which incorporates multiple body segments displays running speed–dependent changes. Specifically, we hypothesize that (1) whole-body dynamic stability is associated with running economy during treadmill running and (2) a singular MLE value incorporating angular velocities from multiple body segments displays running speed–dependent changes. Due to contradictory results presented in previous studies, we have no a priori assumptions about the direction of these relationships.

## Results

**Fig. 1:**
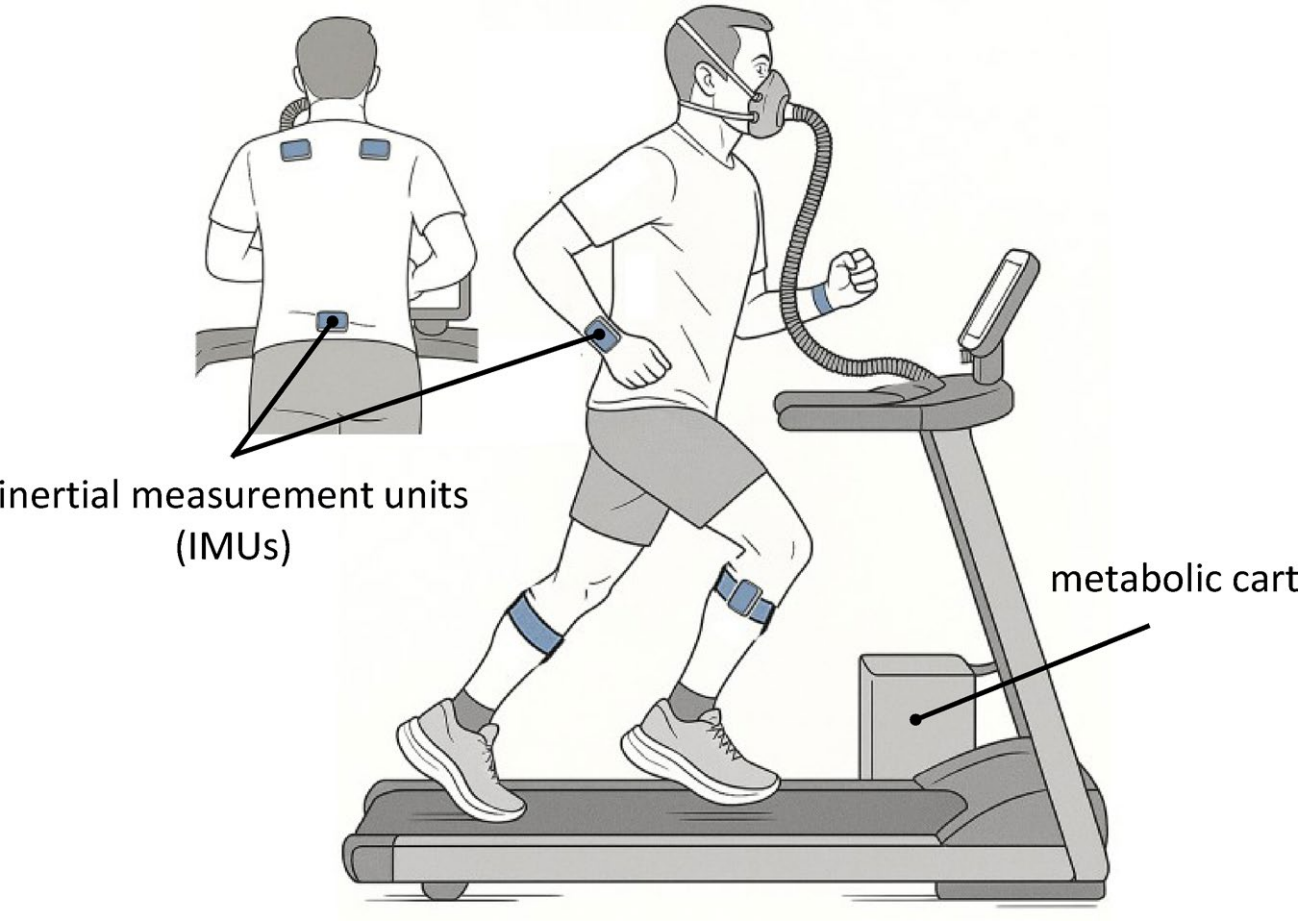
Schematic visualization of the utilized experimental setup, including 7 inertial measurement units attached bilaterally at the shanks, shoulder blades, forearms and at the pelvis as well as a face mask worn by the participants, attached to a metabolic cart for collection of metabolic gas-exchange data.

8 trained female and 11 trained male runners were tested across three individualized running speeds (s_1_ = slowest, s_2_ = medium, s_3_ = fastest) between ventilatory thresholds 1 and 2 on a treadmill. Participants had seven IMUs attached bilaterally at the shanks, shoulder blades, forearms and at the pelvis (Fig. 1), from which 3D angular velocities were combined to a 21D state-space embedding. This multivariate state-space was then used to calculate a singular MLE, which is interpreted as whole-body dynamic stability. Participants were further equipped with a face mask attached to a metabolic cart, which was used to collect metabolic gas-exchange data, from which energy COT was calculated as a running speed–normalized measure of running economy. Results were analyzed using linear mixed models (LMMs) with Tukey-corrected post-hoc comparisons based on estimated marginal means (EMMs).

### Stability effects on cost of transport

**Fig. 2:**
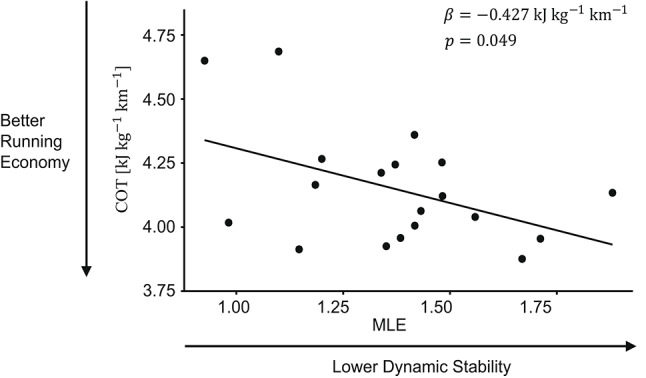
Association between MLE (dynamic stability) and COT (running economy) across participants. Each point represents a single participant, with COT and MLE values averaged over running speeds. *Note*: COT = cost of transport, MLE = maximum Lyapunov exponent.

Complete results from the LMM on dynamic stability and COT are displayed in Table 1. Average MLE across running speeds was negatively associated with COT (*p =* 0.049), indicating that participants with lower dynamic stability tended to have better running economy (Fig. 2). In contrast, within-participant variance in MLE was not associated with COT (*p =* 0.751). The marginal and conditional *R*² values were 0.163 and 0.755, respectively, indicating that the fixed effects accounted for 16.3% of the variance in MLE, with the inclusion of participant-level random effects increasing the explained variance to 75.5%. Of the 16.3% explained variance in COT through fixed effects, 14.4% were explained through the inclusion of between- and within-participant variance in MLE.

**Table 1:** Fixed–effects of the LMM to estimate effects of individualized running speed (categorical) and between- and within-participant variance in mean MLE on COT.

	Estimate	95% CI	SE	DF	t-value	*p*-value
(Intercept)	4.749	[4.202, 5.296]	0.280	17	16.946	< 0.001***
$\:{MLE}_{B}$	–0.427	[−0.820, −0.034]	0.201	17	–2.121	0.049*
$\:{MLE}_{W}$	0.019	[–0.096, 0.133]	0.059	263	0.318	0.751
$\:{s}_{2}$	−0.064	[–0.101, −0.026]	0.019	263	–3.330	< 0.001***
$\:{s}_{3}$	0.020	[–0.018, 0.058]	0.019	263	1.041	0.299

CI = confidence interval, DF = degrees of freedom, MLE = maximum Lyapunov exponent, $\:{MLE}_{B}$= between-participant variance in MLE, $\:{MLE}_{W}$ = within-participant variance in MLE, SE = standard error, * = *p* < 0.05, ** = *p* < 0.01, *** = *p* < 0.001.

### Running speed effects on cost of transport

**Fig. 3:**
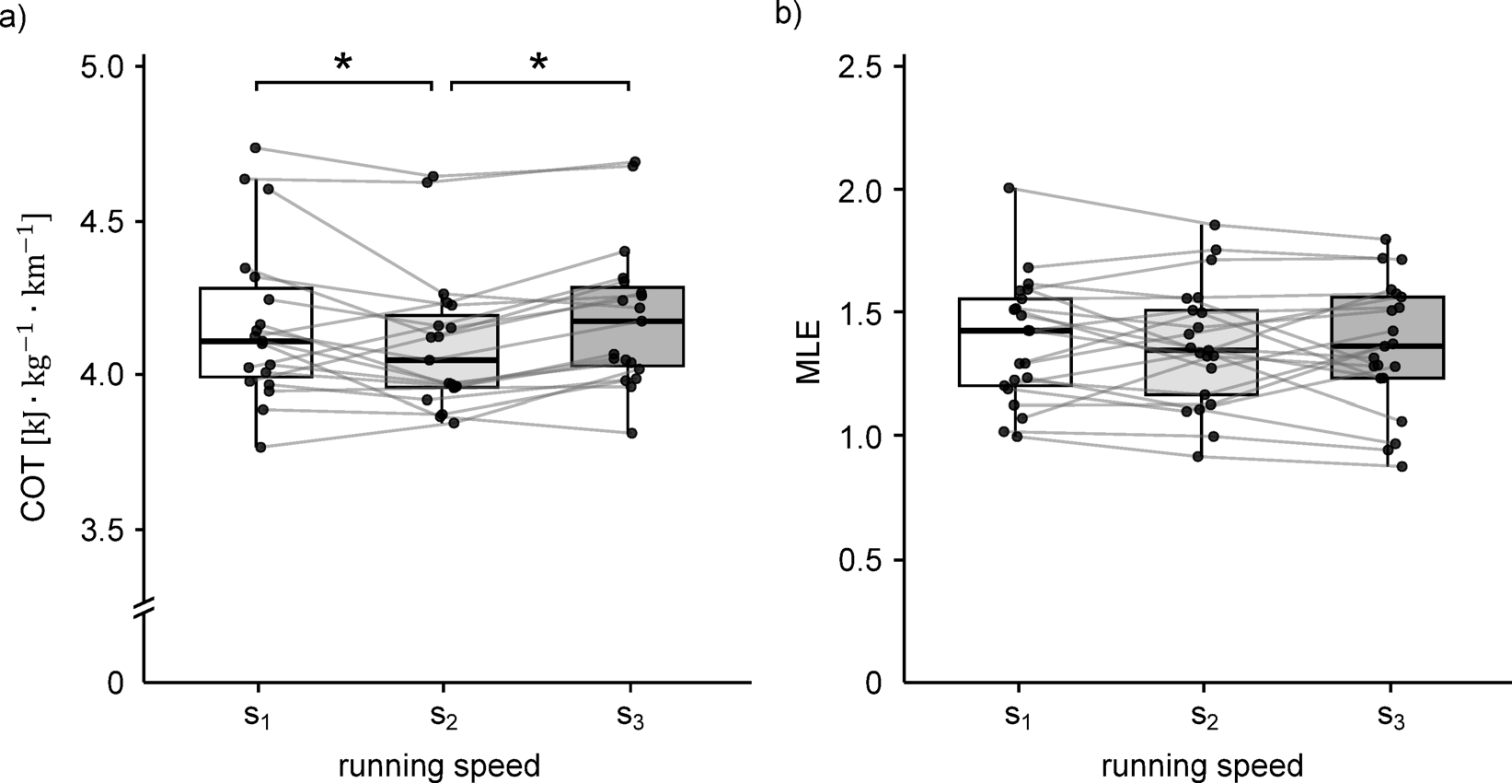
COT (**a**) and MLE (**b**) across running-speed categories. Dots are participant means within each speed and thin grey lines link the same participant across speeds (s₁ = slow, s₂ = medium, s₃ = fast). Boxes show the median (central line) and interquartile range (25th–75th percentiles) and whiskers extend to the most extreme values within 1.5×IQR. Asterisks indicate significant pairwise differences between speed categories (Tukey-adjusted) from a linear mixed-effects model with speed as a fixed effect and participant as a random intercept. COT = cost of transport; MLE = maximum Lyapunov exponent, IQR = interquartile range.

COT increased significantly from $\:{s}_{1}$ to $\:{s}_{2}$ (*p <* 0.001) but not to $\:{s}_{3}$ (*p =* 0.299; Table 1). Pairwise comparisons indicated that COT was significantly lower at $\:{s}_{2}$ relative to $\:{s}_{1}$ (*p =* 0.002) and $\:{s}_{3}$ (*p <* 0.001), whereas there was no difference between $\:{s}_{1}$ and $\:{s}_{3}$ (*p =* 0.484; Fig. 3a). Mean COT values of all trials can be found in Table S1 (supplementary materials) and detailed results of pairwise comparisons of COT between speed categories can be found in Table S2 (supplementary materials).

### Running speed effects on stability

Complete LMM results for the effects of running speed on dynamic stability are presented in Table 2. There were no differences between MLE at speeds $\:{s}_{2}$ (*p =* 0.618) or $\:{s}_{3}$ (*p =* 0.579) compared to MLE at reference speed $\:{s}_{1}$ (Fig. 3b). The marginal and conditional *R*² values were 0.001 and 0.778, respectively, indicating that the fixed effects of categorical running speed accounted for 0.1% of the variance in MLE, with the inclusion of participant-level random effects increasing the explained variance to 77.8%. Mean MLE values of all trials are displayed in Table S3 (supplementary materials).

**Table 2:** Fixed–effects results of the LMM to estimate effects of individualized running speed categories on MLE.

	Estimate	95% CI	SE	DF	t-value	*p*-value
(Intercept)	1.379	[1.270, 1.488]	0.055	27.166	25.023	< 0.001***
$\:{s}_{2}$	–0.018	[–0.090, 0.054]	0.037	40.000	–0.502	0.618
$\:{s}_{3}$	–0.021	[–0.093, 0.051]	0.037	40.000	–0.559	0.579

CI = confidence interval, DF = degrees of freedom, SE = standard error, * = *p* < 0.05, ** = *p* < 0.01, *** = *p* < 0.001.

## Discussion

The primary aim of this study was to investigate the association between whole-body dynamic stability derived from seven different body segments (bilaterally at the shanks, shoulder blades, forearms and at the pelvis) and COT during treadmill running. In support of our primary hypothesis, we found a negative association between dynamic stability and COT (*β =* − 0.427, *p =* 0.049), indicating that lower dynamic stability–assessed in terms of MLE based on a multivariate state-space embedding–is associated with lower COT and thus improved running economy. Contextually, this means that a difference in mean MLE of 1.0 between two runners is associated with a COT difference of ~ 0.43 kJ kg^− 1^ km^− 1^. This difference is meaningful, as a runner with baseline COT of 4.15 kJ kg^− 1^ km^− 1^ (the mean COT in the current dataset) could theoretically lower their COT by ~ 3.0% when moving from the first stability quartile (MLE = 1.192) to the third (MLE = 1.482). Importantly, however, intra-individual variance in COT across trials was not significantly associated with MLE (*p =* 0.751). This suggests that whole-body dynamic stability is a trait-level predictor–rather than state-level predictor–of COT during running and implies that small changes to an individual’s dynamic stability–caused by naturally occurring trial-to-trial or day-to-day variability–are unlikely to affect COT. Therefore, long-term training interventions may be necessary to meaningfully impact COT through changes in trait-level dynamic stability characteristics.

The present study found that the non-linear stability measure of MLE explains 14.4% of inter-individual COT variance in trained runners. While it is the first study to investigate the relationship between MLE and running economy directly, these results are consistent with previous work. For example, Schütte et al.^10^ found that a combination of linear and non-linear stability metrics explained 10.4% in COT variance, with greater mediolateral sample entropy (a measure for signal complexity) being associated with improved running economy. Together, these findings indicate that while other factors explain the majority of COT variance (e.g., metabolic, cardiorespiratory, biomechanical and neuromuscular efficiency; training history)^2^, a small yet functionally relevant portion of running economy is determined by a runner’s dynamic stability characteristics. Counterintuitively, however, more chaotic (i.e., higher MLE; current study), more complex (i.e., higher sample entropy)^10^, less orbitally stable (i.e., higher Floquet multipliers)^11^ and more variable (i.e., higher anteroposterior acceleration root mean square)^10^ movement dynamics are associated with a more efficient running gait. Thus, current evidence suggests that lower dynamic stability is associated with better running economy and indicates that there exists a trade-off between dynamic stability and movement efficiency.

Maintaining dynamic stability during running is associated with distinct energy costs^1^. It is, therefore, likely that a more dynamically stable running technique would be achieved through allocation of additional metabolic energy to the task of stabilization, which would increase metabolic energy expenditure necessary to travel a given distance. Mechanistically, greater dynamic stability may in part be achieved through increased muscular co-activation, which serves to increase joint stability during locomotion^16^ and results in increased muscular activation necessary to produce a given net joint moment. As such, longer stance-phase coactivation of several quadriceps–hamstrings muscle pairs (rectus femoris–biceps femoris, vastus lateralis–biceps femoris, rectus femoris–biceps femoris and rectus femoris–gastrocnemius medialis) is associated with increased COT^17,18^, demonstrating that there exists a tradeoff between joint stability and COT. This tradeoff may mediate the relationship between whole-body dynamic stability and COT found in the present study. For optimal running performance, it may, therefore, be beneficial to reduce dynamic stability control to the lowest level at which stable and safe locomotion can still be ensured, while excessive stability would exert a metabolic penalty and lead to a worsening in running economy. However, further research including electromyographic measurements of agonist–antagonist muscle pairs in addition to measures of dynamic stability and running economy is necessary to elucidate the relationship between muscular coactivation, joint (e.g., ankle, knee, hip), segment (e.g., shank, thigh, lower trunk, upper trunk) and whole-body dynamic stability and running economy.

The secondary aim of the present study was to investigate whether running speed affects the MLE derived from a multivariate state-space embedding which incorporates multiple body segments. Contrary to our second hypothesis, running speed had no effect on MLE (*p >* 0.579), indicating that this singular, whole-body measure of dynamic stability is robust against changes in running speed. These findings contrast previously published results that demonstrate clear running speed effects on local dynamic stability of knee and hip joint as well as center-of-mass dynamics^13–15^. However, these studies are contradictory with regards to the directionality of this effect. Mehdizadeh et al.^14^ and Look et al.^15^ found that dynamic stability of an optical trunk marker as well as knee, hip joint and center-of-mass, respectively, decreased with increasing running speed, while Cerrito et al.^13^ showed an increase in dynamic stability of the hip joint with increasing running speed. Preliminary analyses of our own data add to this contradiction, indicating that when MLE is calculated for individual body segments, running speed–dependent changes are present in both directions. While increasing speed led to increases in stability of the pelvis, upper trunk and forearms, it led to a reduction in stability at the shank (for further details see Tables S4 and S5, supplementary materials). These differences in directionality between previously published works and our data may stem from variations in participant populations (novice vs. experienced runners) as well as in signal types used for dynamic stability calculation (marker positions or joint angles vs. angular velocity). Notably, when calculating MLE based on a multivariate state-space embedding across multiple body segments, these speed–dependent changes disappear with neither individualized running speed categories (i.e., slow, medium, fast; presented in the results section) nor absolute running speed (i.e., in km/h; Table S6, supplementary materials) affecting MLE. This indicates that certain signal characteristics that are present at specific body segments may be masked by combination of segmental angular velocities to a single state-space. Still, the resulting measure appears to be robust to changes in running speed, making it feasible to assess and compare between individuals running at different speeds. This provides a key benefit of the multivariate MLE when compared to values derived from individual body segments, which should only be assessed and compared at a single speed due to their speed dependencies.

A more general issue of dynamic stability studies in running is the fact that many studies have shown that dynamic stability as measured by the MLE is affected by demographic, training and environmental characteristics (e.g., sex^19^, running experience^4,8^, fatigue^4^, etc.) but what is typically not evident from these studies is the specific way in which the found results are practically relevant for runners or other stakeholders of the sport. To show practical relevance, a direct link between the MLE and parameters relevant to injury risk or performance first needs to be demonstrated, which–to the knowledge of the authors–has not yet happened. Here, for the first time we demonstrate that dynamic stability can be directly linked to running economy, a measure that is highly relevant for running performance^20,21^. Future studies should focus on establishing practical relevance of non-linear measures of dynamic stability rather than simply comparing measures across different conditions and populations.

Finally, there are several limitations to the present study that need to be acknowledged. For one, while we preserved the coupled dynamics of the locomotor system through combining 3D angular velocities of different body segments into one multivariate state-space, the resulting estimate of whole-body dynamic stability may still fall short of fully capturing the stability of the complex, multi-degree-of-freedom system that is human running. Future work should focus on elucidating the systematic effects of incorporating multiple body segments into dynamic stability calculations and determine the optimal placements of IMUs to determine whole-body dynamic stability. Further, measures of whole-body dynamic stability might be validated using artificially imposed balance deficits, which has recently been successfully applied to walking^22^ and may offer a method of objectively determining effectiveness of running stability measures.

Additionally, while treadmill running in a laboratory environment greatly simplifies data collection and allows for highly controlled measurements, there are kinematic and kinetic differences between treadmill and overground running and results from one cannot simply be generalized to the other^23^. Lastly, the present study as well as most previous research on dynamic stability in running focused on controlled environments and smooth surfaces (e.g., laboratory treadmills, road and track surfaces)^3^, but stability may be more relevant during uneven running conditions (e.g., trail running), where the presence of frequent perturbations demands greater neuromuscular control to maintain stable locomotion^24,25^. This may help to explain why traditional predictors of endurance performance–such as $\:\dot{V}{O}_{{2}_{max}}$, $\:\dot{\%V}{O}_{{2}_{max}}$at ventilatory threshold and running economy–fail to fully account for trail running performance^26^. Thus, future research should explore whether dynamic stability during uneven running conditions may explain a larger portion of inter-individual variance in running economy.

## Conclusions

This study is the first to directly link whole-body dynamic stability, assessed via a multivariate maximum Lyapunov exponent, to running economy in trained runners. By embedding angular velocity signals from seven body segments into a unified state-space, we demonstrated that runners with lower dynamic stability exhibited lower cost of transport, indicating superior running economy. Importantly, this relationship emerged at the inter-individual level only, suggesting that whole-body dynamic stability behaves more as a stable trait than a transient state. Small, trial-to-trial fluctuations in stability within individuals were not associated with energetic cost, implying that meaningful changes in running economy may require long-term adaptations in stability-related control strategies rather than acute adjustments. Contrary to expectations, multivariate MLE was unaffected by running speed across three individualized intensities. This robustness distinguishes whole-body stability from segment-level measures, which have been shown to vary with speed. Collectively, our findings support that dynamic stability may be a subtle but functionally relevant determinant of running economy, explaining ~14% of inter-individual COT variance through dynamic stability. Rather than more stable movement being universally advantageous, our results support a trade-off in which energetic efficiency may be optimized by a degree of dynamic instability. This challenges traditional interpretations of stability as inherently beneficial and suggests that economical runners may exploit controlled variability to minimize the metabolic costs of over-stabilization.

## Methods

### Subjects

Twelve male (34.9 ± 8.3 years, 71.1 ± 8.5 kg, BMI 22.4 ± 1.9 $\:kg\:{m}^{-2}$, $\:\dot{V}{O}_{{2}_{peak}}$62.0 ± 5.1 $\:mL\:{kg}^{-1}\:{min}^{-1}$) and nine female (29.1 ± 10.2 years, 62.3 ± 5.9 kg, BMI 21.8 ± 1.5 $\:\text{k}\text{g}\:{\text{m}}^{-2}$, $\:\dot{V}{O}_{{2}_{peak}}$52.2 ± 3.2 $\:m\text{L}\:{\text{k}\text{g}}^{-1}\:{\text{m}\text{i}\text{n}}^{-1}$) trained runners participated in this study. All participants had a minimum weekly training volume of 20 km (average 32.4 ± 12.0 km) and a recorded seasonal 10 km race time under 45 min for males (36:50 ± 2:50 min) and 50 min for females (45:06 ± 3:50 min). On the day of testing, all runners reported being in good health and free from musculoskeletal injuries for at least three months. Written informed consent was obtained from all participants prior to their involvement. The study adhered to the principles of the Declaration of Helsinki and received ethical approval from the Ethics Committee of the Department of Engineering and Industrial Design at Magdeburg-Stendal University of Applied Sciences (certificate number EKIWID-2023-09-001RM).

### Experimental protocol

A randomized cross-over design was used to evaluate the effects of dynamic stability on COT as well as the running speed effects on dynamic stability. For this, runners were tested on a treadmill across a comprehensive range of individualized running speeds between first and second ventilatory thresholds. Participants attended two separate lab sessions. In the first session, participants completed an incremental test on a motorized treadmill (Star Trac FreeRunner 10TRx, Core Health 6 Fitness, Vancouver, BC, Canada), starting at 6.0 $\:\text{k}\text{m}\:{\text{h}}^{-1}$ with 3 min stages increasing by 2.0 $\:\text{k}\text{m}\:{\text{h}}^{-1}$ until voluntary exhaustion was reached, while breath-by-breath pulmonary gas exchange data were collected using a metabolic cart (MetaMax 3B, CORTEX Biophysik GmbH, Leipzig, Germany) and used to determine running speeds at ventilatory thresholds 1 and 2 (sVT1 and vVT2). $\:\dot{V}{O}_{{2}_{peak}}$was defined as the maximum oxygen consumption recorded during this session.

The aim of the second visit was to assess dynamic stability and COT over a range of running speeds from easy running to race pace. During this session, each participant performed five trials of 3 × 3 min of treadmill running at the three individual running speeds $\:{s}_{1}$ = 90% sVT1, $\:{s}_{2}$=$\:\:\frac{1}{2}\:$(sVT1+sVT2), and $\:{s}_{3}$= 100% sVT2. The present dataset was collected as part of a larger project which incorporated testing of four different advanced footwear technology running shoe models against each runner’s own habitual training shoes, which is why participants wore a different pair of shoes for each bout in randomized order (for details see Riedl et al.^27^. For the purpose of the present study, dynamic stability and COT were averaged across footwear conditions. The associated intensities of “easy” (*s*_*1*_), “threshold” (*s*_*2*_), and “competition” (*s*_*3*_) were chosen as they represent speeds that are typically used by runners in training and racing. Average running speeds at $\:{s}_{1}$, $\:{s}_{2}$ and $\:{s}_{3}$ were 10.31 ± 0.96, 13.43 ± 1.38 and 15.39 ± 1.75 $\:\text{k}\text{m}\:{\text{h}}^{-1}$, respectively. Each trial consisted of 3 consecutive 3 min intervals of increasing speed at *s*_*1*_, *s*_*2*_ and *s*_*3*_ in fixed order, while breath-by-breath pulmonary gas exchange data were collected. The 3 min interval duration was chosen to ensure metabolic steady-state while minimizing fatigue accumulation^28^. Additionally, metabolic steady state was confirmed visually (continuous COT data from all subjects across all trials as well as individual running speeds are displayed in Fig. S1, supplemental materials), and limited anaerobic contribution was assured by a respiratory exchange ratio (RER) of < 1.0 during all trials. 3D angular velocities were recorded using seven inertial measurement units (IMUs; 100 Hz; Xsens Awinda, Xsens Technologies, Enschede, the Netherlands), which were attached directly to the participants’ skin using a combination of proprietary straps and sports tape at the following locations: left and right tibiae, sacrum, left and right shoulder blades and left and right radii. The full experimental setup is displayed in Fig. 1. Treadmill velocity was automatically controlled by the spirometry software through a participant-specific protocol and written into the metabolic cart data file, synching metabolic data to treadmill velocity. IMU data collection was then manually synched to metabolic data by beginning and ending recording at 30 s and 180 s, respectively, of running at a given speed. Each trial was separated by five minutes of rest to allow for shoe change and sufficient recovery, which was verified by heartrate and cardiopulmonary values prior to starting the subsequent trial. Runners were instructed to abstain from strenuous exercise for at least 48 h prior to both laboratory visits and were encouraged to match diet and sleep patterns as closely as possible between visits 1 and 2.

### Data processing and analysis

#### Cost of transport

Ventilatory thresholds 1 and 2 were determined by two independent experts using the Cortex MetaSoft software suite 5.5.1 before exporting the dataset for further analysis. Further data processing steps and analyses of cardiopulmonary data were conducted using a custom script written in MATLAB R2023a (The MathWorks Inc., Natick, MA, USA). All cardiopulmonary data were first cleaned by removing outliers that exceeded the mean of a 7-breath window by more than 2 standard deviations and then smoothed by applying a 7-breath moving average^29^. Cardiopulmonary datasets from visit 2, which consist of 5 trials (one per footwear condition) and 3 intervals per trial (one per speed), were split into individual intervals, so that for each participant 5 × 3 = 15 intervals of gas exchange data were used for further analysis. From these 15 blocks of 3 min intervals, the final 60 s of $\:\dot{V}O2$ and $\:\dot{V}CO2$ data were averaged and used to calculate energy consumption by use of Pérronet and Massicotte’s non-protein respiratory quotient equations^30^. Mean COT (kJ kg^− 1^ km^− 1^) was then calculated to normalize energy consumption with respect to body mass and running speed. Two runners (subjects 15 & 19; 1 male, 1 female) exceeded RER of 1.0 during their $\:{s}_{3}$ intervals and thus were excluded from all COT analyses of the present study, reducing the sample to 19 runners (11 male, 8 female).

#### Whole-body dynamic stability

All data processing and analysis for dynamic stability were conducted in Python (3.11.9). To quantify dynamic stability, short-term maximum Lyapunov exponents (MLE) were computed from a high-dimensional state space constructed from all seven IMUs, attached bilaterally on the shanks, forearms, shoulder blades, and at the pelvis. From each device, the three axes of angular velocity were used, resulting in a 21-dimensional signal at each time sample. Initial contacts of the right foot were identified using angular velocity of the right shank about the mediolateral axis^31,32^. The final 100 strides of each 3-min interval were extracted and time-normalized to 10,000 points (100 strides × 100 samples per stride)^3^. To analyze the signals, a 21-dimensional state-space vector $\:s\left(t\right)$ was reconstructed using time-delayed embedding as follows:

1 $$\:s\left(t\right)=[x\left(t\right),x\left(t+{\uptau\:}\right),x\left(\text{t}+2{\uptau\:}\right)\dots\:,x\left(\text{t}+\left({d}_{E}-1\right){\uptau\:}\right)]\:$$

where $\:x\left(t\right)$ is the concatenated 21-dimensional vector of time-normalized gyroscope signals, τ is the time delay, and *d*_*E*_ is the embedding dimension. Time delay (*τ*) was selected from the first minimum of the average mutual information, and embedding dimension (*d*_*E*_) was determined using the global false nearest neighbors method^3^. Integer mean values of τ and *d*_*E*_ across participants and conditions were used for final embedding (*τ =* 8, *d*_*E*_ *=* 2).

Divergence curves were calculated using the Rosenstein algorithm^33^. For each state vector, the nearest non-temporal neighbor was identified using a Theiler window of one stride to avoid autocorrelation. Logarithmic divergence was tracked over time, and the short-term slope (visually identified as 25% of one stride^34^ of the divergence curve defined MLE (for exemplary plots see Fig. S2, supplemental materials)). Higher MLE indicates faster divergence of trajectories and therefore lower dynamic stability. Unlike prior approaches that calculated separate MLE values for individual body segments, this method quantifies whole-body stability from a unified, high-dimensional representation of running dynamics, preserving the coupled dynamics of the locomotor system. Due to minimum time-series length requirements of the MLE for state-space reconstruction, it was not possible to synch MLE values directly to COT values, as COT could only be validly determined during the final 60 s of running at a given speed^28^, which was shorter than the time period it took runners to complete the requisite number of strides. However, based on average stride frequencies of 1.34, 1.40 and 1.45 strides $\cdot$ min^− 1^ during s_1_, s_2_ and s_3_, respectively, MLE was assessed during the final 74.53, 71.43 and 68.97 s, resulting in only a short time period during which data was collected for MLE calculation but not for COT calculation.

### Statistical analysis

#### Stability and cost of transport

All statistical analyses were performed in R Studio (version 2024.04.2; RStudio PBC, Boston, MA, USA). To investigate whether dynamic stability quantified via MLE is associated with COT, we utilized a linear mixed-effects model with a decomposition of within- and between-participant effects. This approach allowed for separate investigation of whether within- or between-participant variance in MLE was associated with a systematic change in COT. In essence, we could evaluate whether participants’ average MLE values (averaged across all running trials; between-participant differences) or participants’ trial-to-trial deviations from their own average MLE (within-participant variability) were associated with corresponding systematic changes in COT. To achieve this, we first calculated the participant-specific average MLE across all running trials to capture between-participant variation, and then calculated the deviation from this mean on each trial to isolate within-participant variation. This group-mean centering ensures that within- and between-participant effects are evaluated separately and that these effects are not conflated. The model was estimated using restricted maximum likelihood (REML) and had the following form:

2 $$\:{COT}_{ij}={\beta\:}_{0}+{\beta\:}_{B}\cdot\:{\stackrel{-}{X}}_{j}+{\beta\:}_{W}\cdot\:\left({X}_{ij}-{\stackrel{-}{X}}_{j}\right)+{\beta\:}_{2}\cdot\:{Z}_{ij}+{u}_{j}+{\varepsilon\:}_{ij}$$

where $\:{COT}_{ij}$ is the dependent variable for trial *i* of participant *j*, $\:{X}_{ij}$ is the trial-level predictor (i.e., MLE during trial *i* of participant *j*), $\:{\stackrel{-}{X}}_{j}$ is the participant-specific mean MLE across all trials (between-participant component), $\:{X}_{ij}-{\stackrel{-}{X}}_{j}$ is the deviation from a participant’s own mean MLE (within-participant component) and $\:{Z}_{ij}$ are additional fixed-effects covariates (i.e., speed). $\:{\beta\:}_{0}$ is the fixed intercept (expected COT when all predictors are at reference levels), $\:{\beta\:}_{B}$ is the between-participant slope (indicating whether participants with higher average MLE values tend to have higher average COT), $\:{\beta\:}_{W}$ is the within-participant slope (indicating whether a participant’s COT increases on trials where their MLE is higher than usual) and $\:{\beta\:}_{2}$ are fixed effect coefficients for the additional covariates. Finally, $\:{u}_{j}\:\sim\:N(0,{\sigma\:}_{u}^{2})$ is the random intercept for participant *j* and $\:{\varepsilon\:}_{ij}\:\sim\:N(0,{\sigma\:}^{2})$ is the residual error term for trial *i*. Marginal and conditional *R*^*2*^ were determined to evaluate model fit and assumptions of linearity, homoscedasticity and normality of residuals as well as for potential participant-specific deviations were tested.

Marginal *R*^*2*^ of this model (incl. MLE predictors) was compared to a simpler model which only included fixed-effects for speed to determine the amount of additional variance explained through inclusion of MLE predictors. EMMs for running speed were obtained by averaging over footwear conditions and pairwise comparisons were performed using Tukey adjusted tests.

#### Stability and running speed

To test whether running speed influences dynamic stability, we first aggregated MLE within participant and speed category, averaging across all footwear conditions to isolate speed effects. We fit an LMM with speed as a categorical predictor (levels: s_1_, s_2_, s_3_; reference: s_1_) and a random intercept for participant to account for repeated measures. The model was estimated via REML:

3 $$\:{MLE}_{ij}=\:{\beta\:}_{0}+\:{{\beta\:}_{S}}^{\text{T}}{S}_{ij}+{u}_{j}+{\varepsilon\:}_{ij}\:$$

where $\:{MLE}_{ij}$ is the multivariate MLE for trial *j* of participant $\:i\:(i\:=\:1,\dots\:,N)\text{}\:$is the fixed intercept (expected MLE at the reference speed category s_1_), $\:{\beta\:}_{S}$​ is a vector of fixed effects associated with the non-reference speed categories (s_2_ and s_3_) and $\:{S}_{ij}$ is the corresponding design vector encoding the trial’s speed. $\:{u}_{j}\sim\:N(0,{\sigma\:}_{u}^{2})$ is the random intercept for participant *j* and $\:{u}_{j}\sim\:N\left(0,{\sigma\:}^{2}\right)$ is the residual error term. Explanatory power of the linear mixed–effects model was assessed using marginal *R*² (variance explained by the fixed effects alone) and conditional R² (variance explained by both fixed and random effects) values. These indices provide a measure of overall model fit and an assessment of the relative contribution of predictors and the random intercept for participant. The final LMM was evaluated for assumptions of linearity, homoscedasticity and normality of residuals as well as for potential participant-specific deviations that could influence the overall fit. Results are presented as means ± standard deviation and a priori alpha level was set at *p* = 0.05 for all tests.

## Supplementary Information

Below is the link to the electronic supplementary material.

Supplementary Material 1

## Data Availability

All experimental data, alongside scripts for data processing and statistical analyses, are available from the corresponding author on request.
