# Supplementary material for: Runners with lower dynamic stability exhibit better running economy
Source: Sci Rep. 2025 Oct 31;15:38117. doi: 10.1038/s41598-025-26008-x (PMC12579230; doi:10.1038/s41598-025-26008-x)
Supplement: Supplementary file 1 — Supplementary Material 1 [file 41598_2025_26008_MOESM1_ESM.pdf]

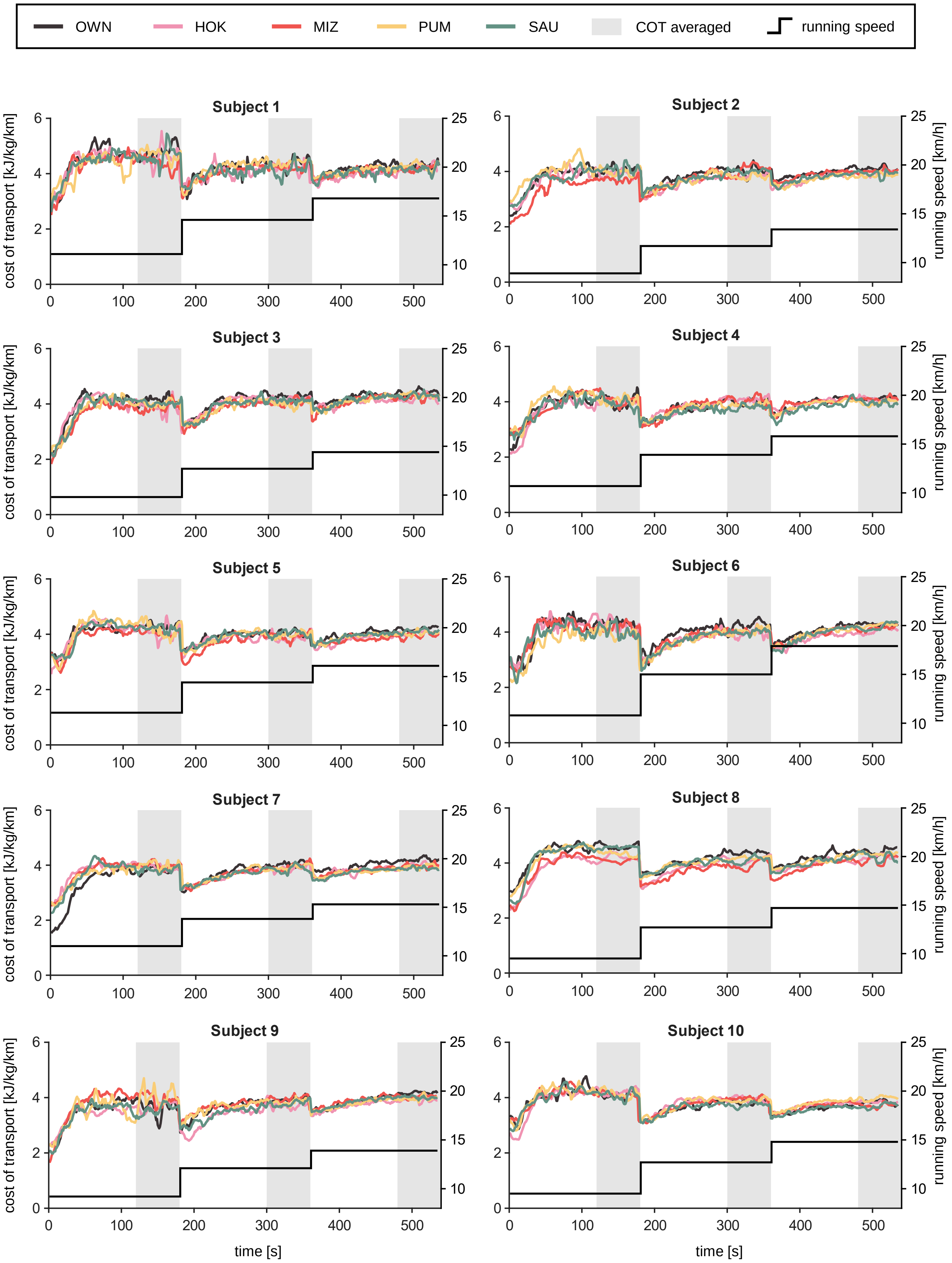

OWN
HOK
MIZ
PUM
SAU
COT averaged
running speed
Subject 1
Subject 2
Subject 3
Subject 4
Subject 5
Subject 6
Subject 7
Subject 8
Subject 9
Subject 10
cost of transport [kJ/kg/km]
running speed [km/h]
time [s]

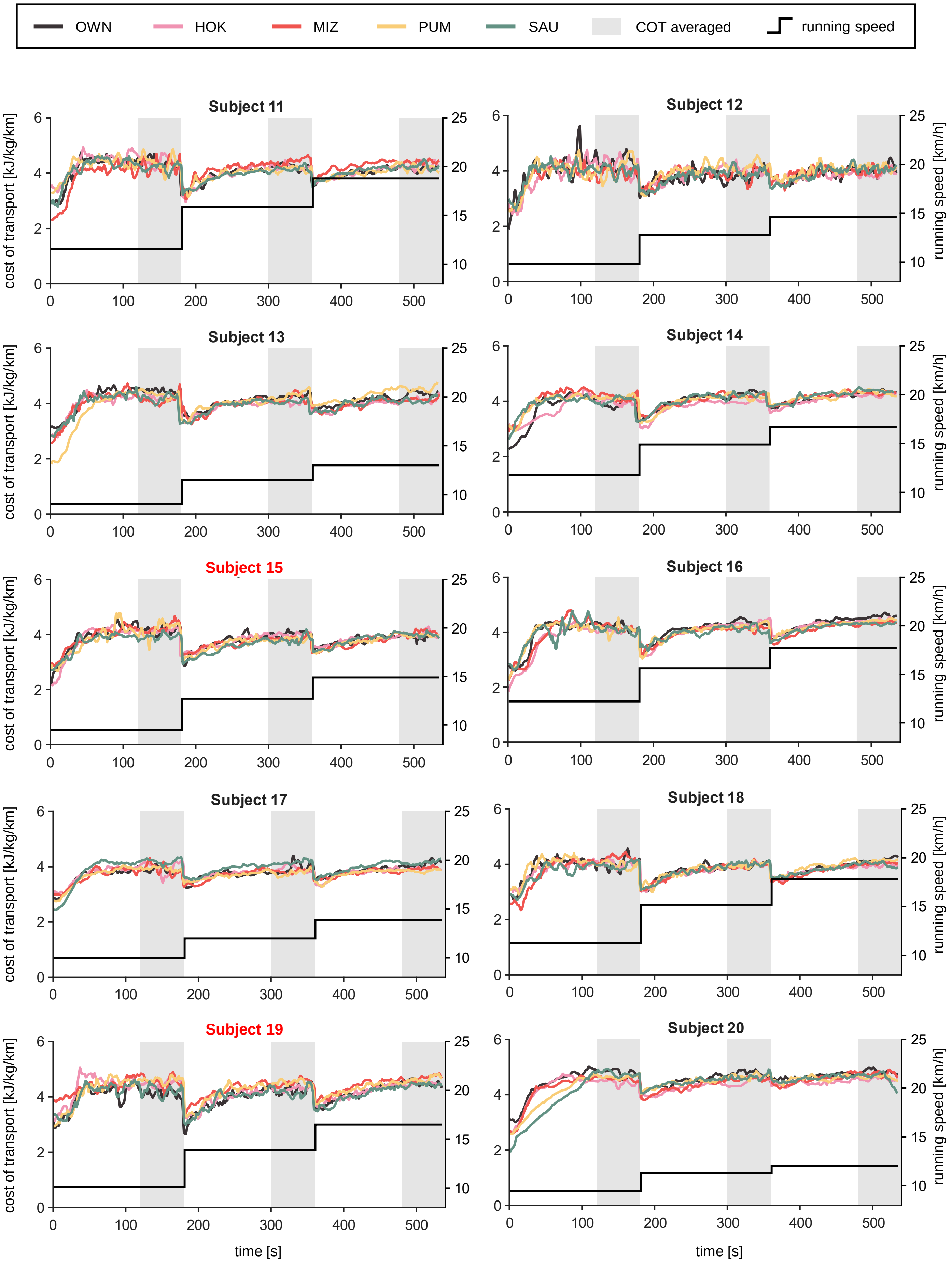

OWN
HOK
MIZ
PUM
SAU
COT averaged
running speed
Subject 11
Subject 12
Subject 13
Subject 14
Subject 15
Subject 16
Subject 17
Subject 18
Subject 19
Subject 20
cost of transport [kJ/kg/km]
running speed [km/h]
time [s]

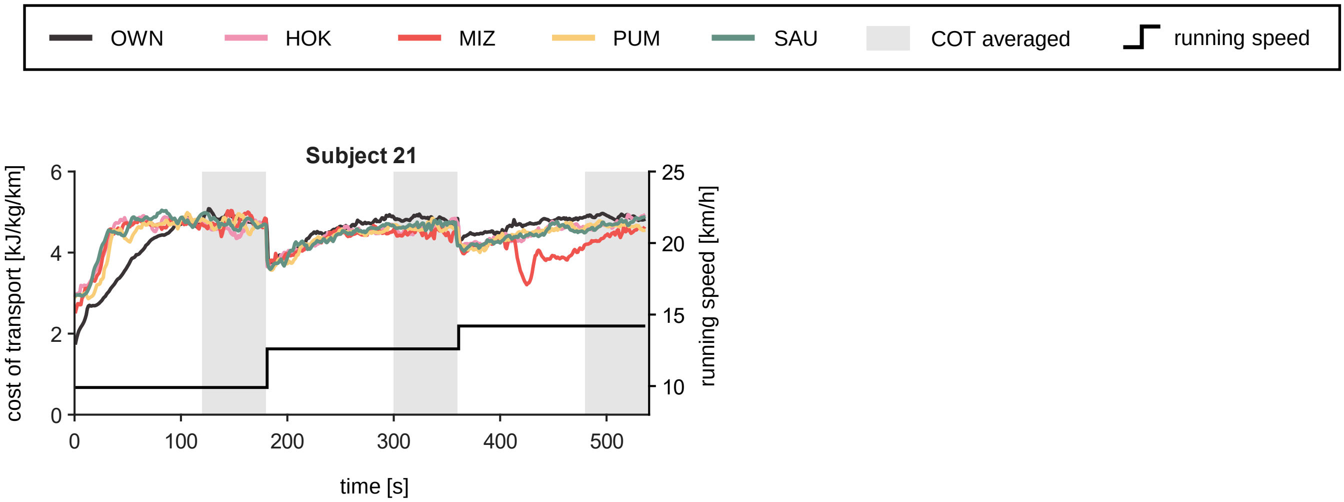

Fig. S1 Continuous cost of transport data from all subjects across all footwear conditions (left y-axis) and running speed (right y-axis). *Note:* Subjects 15 and 19 were removed from cost of transport analysis due to exceeding RER of 1.0 during the fastest running speed.

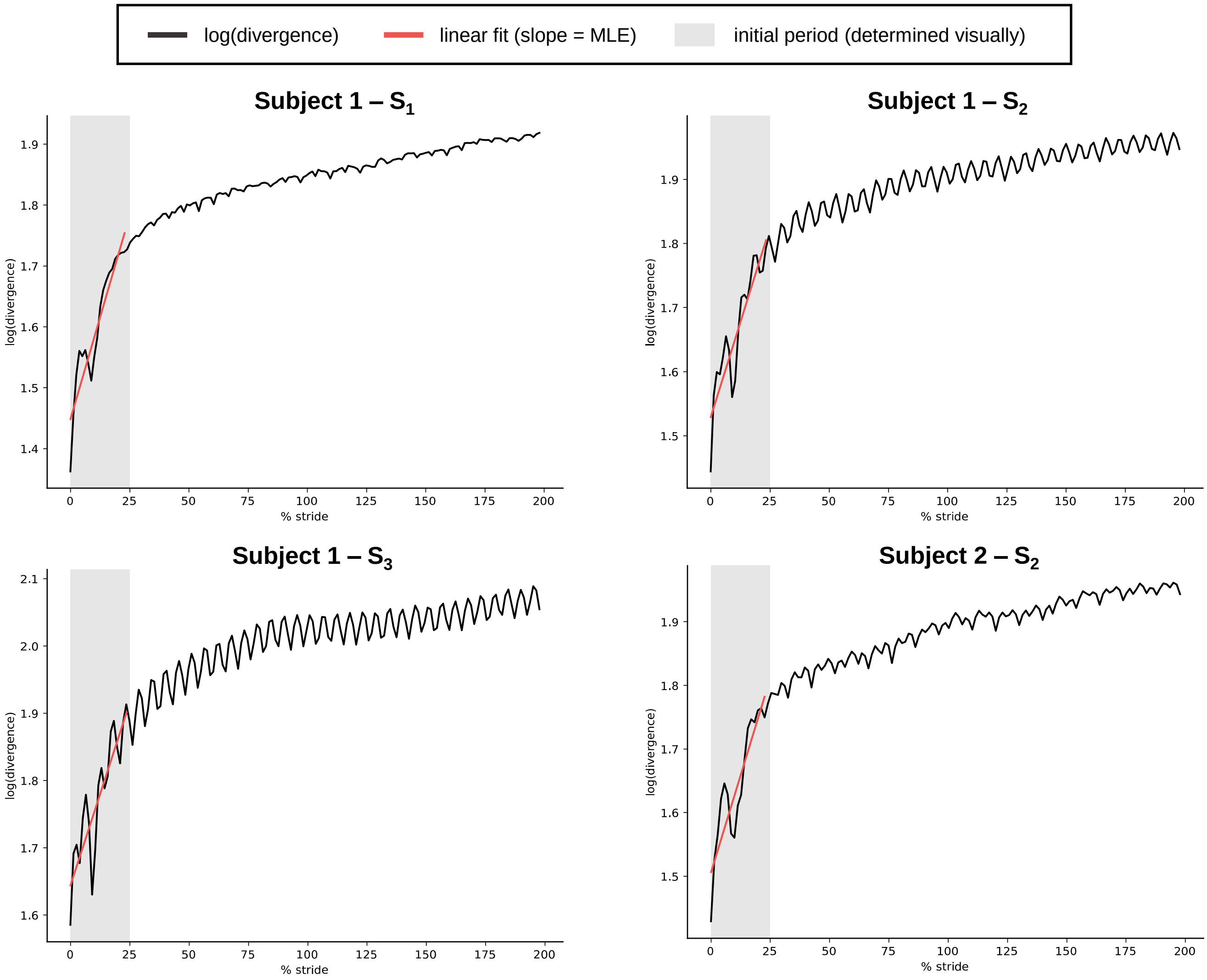

Fig. S2 Exemplary plots of logarithmic divergence of initially neighboring points in state-space (black) and linear fits (red) through the initial phase of divergence. The slope of the linear fit equals the maximal Lyapunov exponent. *Note:* S_1 = slowest speed, S_2 = medium speed, S_3 = fastest speed.

Table S1. COT values at each combination of footwear condition and running speed.

Condition	Speed Category	COT [$kJ \cdot kg^{-1} \cdot km^{-1}$, mean±SD]	
OWN	s1	4.23	0.34
OWN	s2	4.17	0.28
OWN	s3	4.28	0.26
HOK	s1	4.13	0.25
HOK	s2	4.06	0.23
HOK	s3	4.14	0.23
MIZ	s1	4.13	0.25
MIZ	s2	4.09	0.21
MIZ	s3	4.14	0.23
PUM	s1	4.19	0.26
PUM	s2	4.10	0.26
PUM	s3	4.20	0.25
SAU	s1	4.15	0.31
SAU	s2	4.07	0.25
SAU	s3	4.16	0.25

Note: HOK = Hoka Rocket X2, MIZ = Mizuno Wave Rebellion Pro, PUM = Puma S3-R Nitro Elite, SAU = Saucony Endorphin Pro 3, s_1 = slowest speed, s_2 = medium speed, s_3 = fastest speed.

Table S2. Pairwise comparisons of COT between speed categories. Comparisons are based on estimated marginal means and p-values are adjusted based on Tukey's method for comparing a family of 3 estimates.

Contrast	Estimate	SE	DF	t-ratio	p-value	
s1-s2	0.063	0.018	259	3.507	0.002	**
s1-s3	–0.021	0.018	259	–1.150	0.484	
s2-s3	–0.085	0.018	259	–4.651	<0.001	***

Note: SE = standard error, DF = degrees of freedom, s_1 = slowest speed, s_2 = medium speed, s_3 = fastest speed, * = $p < 0.05$, ** = $p < 0.01$, *** = $p < 0.001$.

Table S3. MLE values at each combination of footwear condition and running speed.

Condition	Speed Category	MLE (mean±SD)
OWN	s1	1.35 ± 0.24
OWN	s2	1.35 ± 0.25
OWN	s3	1.35 ± 0.27
HOK	s1	1.43 ± 0.29
HOK	s2	1.37 ± 0.27
HOK	s3	1.40 ± 0.29
MIZ	s1	1.34 ± 0.26
MIZ	s2	1.32 ± 0.24
MIZ	s3	1.37 ± 0.24
PUM	s1	1.37 ± 0.28
PUM	s2	1.35 ± 0.28
PUM	s3	1.32 ± 0.32
SAU	s1	1.40 ± 0.27
SAU	s2	1.40 ± 0.24
SAU	s3	1.35 ± 0.27

Note: HOK = Hoka Rocket X2, MIZ = Mizuno Wave Rebellion Pro, PUM = Puma S3-R Nitro Elite, SAU = Saucony Endorphin Pro 3, s_1 = slowest speed, s_2 = medium speed, s_3 = fastest speed.

Table S4. Results from a preliminary analysis that included footwear condition and a single MLE value per body segment. Fixed–effects of the LMM to estimate effects of footwear condition, speed and sensor location on MLE indicate speed effects on MLE.

	Estimate	95% CI	SE	DF	t-value	p-value	
(Intercept)	0.642	[0.599, 0.684]	0.022	643	29.49	< 0.001	***
HOK	0.010	[-0.002, 0.021]	0.006	1228	1.669	0.0955	
MIZ	0.008	[-0.004, 0.019]	0.006	1228	1.339	0.1809	
PUM	0.011	[0.000, 0.022]	0.006	1228	1.879	0.0604	

SAU	0.005	[-0.006, 0.017]	0.006	1228	0.917	0.3592	
Speed	-0.003	[-0.006, -0.001]	0.002	1240	-2.292	0.0221	*
Sensor location (forearm)	-0.149	[-0.202, -0.096]	0.027	1228	-5.489	< 0.001	***
Sensor location (upper trunk)	-0.220	[-0.273, -0.167]	0.027	1228	-8.107	< 0.001	***
Sensor location (shank)	0.035	[-0.018, 0.088]	0.027	1228	1.284	0.1993	
Speed×Sensor location (forearm)	-0.003	[-0.007, 0.001]	0.002	1228	-1.344	0.1793	
Speed×Sensor location (upper trunk)	-0.002	[-0.006, 0.002]	0.002	1228	-0.796	0.4262	
Speed×Sensor location (shank)	0.010	[0.006, 0.014]	0.002	1228	4.677	< 0.001	***

Note: CI = confidence interval, SE = standard error, DF = degrees of freedom, HOK = Hoka Rocket X2, MIZ = Mizuno Wave Rebellion Pro, PUM = Puma S3-R Nitro Elite, SAU = Saucony Endorphin Pro 3, * = $p < 0.05$, ** = $p < 0.01$, *** = $p < 0.001$.

Table S5. Results from a preliminary analysis that included footwear condition and a single MLE value per body segment. Pairwise comparisons of speed trends of MLE between sensor locations show that body segment–specific MLE values are affected differently by an increase in running speed. Comparisons are based on estimated marginal means of linear trends and p-values are adjusted based on Tukey’s method for comparing a family of 3 estimates.

**Contrast**	**Estimate**	**SE**	**DF**	**t-ratio**	**p-value**	
pelvis-forearm	0.003	0.002	1228	1.344	0.535	
pelvis-upper trunk	0.002	0.002	1228	0.796	0.856	
pelvis-shank	-0.010	0.002	1228	-4.677	<.001	***
forearm-upper trunk	-0.001	0.002	1228	-0.548	0.947	
forearm-shank	-0.012	0.002	1228	-6.021	<.001	***
upper trunk-shank	-0.011	0.002	1228	-5.473	<.001	***

Note: SE = standard error, DF = degrees of freedom, * = $p < 0.05$, ** = $p < 0.01$, *** = $p < 0.001$.

Table S6. Fixed–effects of the LMM to estimate effects absolute running speed (in km·h^{-1}) on MLE.

	**Estimate**	**95% CI**	**SE**	**DF**	**t-value**	**p-value**	
(Intercept)	1.366	[1.208, 1.611]	0.103	60.051	13.727	< 0.001	***

absolute running speed	–0.008	[-0.017, 0.010]	0.007	43.690	–0.483	0.631

Note: CI = confidence interval, SE = standard error, DF = degrees of freedom, * = $p < 0.05$, ** = $p < 0.01$, *** = $p < 0.001$.
